# Mortality and Morbidity of Beef Calves in Free-Range Farms in Alentejo, Portugal—A Preliminary Study

**DOI:** 10.1155/2019/3616284

**Published:** 2019-10-16

**Authors:** Rute Santos, Ana Cachapa, Graça P. Carvalho, Carolina B. Silva, Laura Hernández, Lina Costa, Luísa S. Pereira, Miguel Minas, Helena Vala

**Affiliations:** ^1^Polytechnic Institute of Portalegre, Agrarian School of Elvas, 7350-092 Elvas, Portugal; ^2^VALORIZA—Research Centre for Endogenous Resources Valorization, Polytechnic Institute of Portalegre, 7300-555 Portalegre, Portugal; ^3^Polytechnic Institute of Viseu, Agrarian School of Viseu, 3500-606 Viseu, Portugal; ^4^CITAB—Centre for the Research and Technology of Agro-Environmental and Biological Sciences, University of Trás-os-Montes e Alto Douro, 5001-801 Vila Real, Portugal

## Abstract

Extensive cow-calf beef cattle farms play a significant role in Portuguese livestock production, but records of important production variables, such as calf mortality, are scarce. To assess herd-level calf mortality and its potential economic impact, farmers from the Alentejo region were asked to fill a questionnaire regarding herd size, feeding and reproductive management practices, calf mortality (from birth to weaning), and detection of the main morbidity causes, as well as of sudden, unexplained deaths during the previous 12 months. Farmers were also requested to quantitatively assess the economic impact of calf mortality on their annual revenue. In the majority of farms, the herd size was larger than 100 animals. The median stocking rate was 0.41 adult animals/hectare, and 70% of farmers stated their farm was self-sufficient in forage. The percent of live births that resulted in weaned calves averaged 94.3%, which led to the assumption of a 5.7% calf mortality rate from birth to weaning. In the previous 12 months, 78.8% of the farms identified at least one case of calf diarrhea, 60.7% identified at least one case of respiratory disease, and 82.1% had at least one sudden, unexplained calf death. As expected, farmers that assessed a higher impact of calf losses on their annual economic revenue were also those who reported higher incidences of calf diarrhea, respiratory disease, and sudden, unexplained deaths. One-quarter of the farmers were unable to assess the economic impact of calf mortality on the farm's revenue. Herd size appears to have a predictive value on calf mortality in the first month of life, and from 30 days to weaning. The number of sudden, unexplained calf deaths seems to have a predictive value on overall calf mortality (from birth to weaning), suggesting that the farms' management practices probably play an important role in calf mortality throughout the suckling period. Further studies are needed to fully understand calf mortality in Portuguese extensive rearing systems.

## 1. Introduction

According to the official economic accounts for agriculture in 2016, the value of livestock production in Portugal was 2630.9 million Euros, corresponding to 37.9% of the total value of agricultural production. Cattle were the second main product, representing 21.6% of the overall value, and showed a 5.1% increase from 2015 to 2016. Self-sufficiency in beef production in the European Union is close to 102%; however, in Portugal, this value is only 57% [1], making it the country's meat sector with a greater dependence on imports. Beef cattle farms are mainly located in the south of the country, the Alentejo region, which holds over 65% of the grassland area and around 42% of the cattle stock in Portugal [2]. These cattle are mostly bred in extensive cow-calf production systems, wherein the farmers' revenue is highly dependent on productive efficiency, which is aimed at obtaining one calf per cow, per year. This goal relies mainly on adequate breeding strategies and herd health management, with the latter having a significant effect on pre-weaning calf mortality and morbidity. Calf production is the main goal of beef cattle farming, for raising, fattening, and selling cattle, and rearing replacement heifers to be included in the breeding herd. Loss of young stock due to on-farm mortality is a serious concern affecting the profitability of beef production farms [3].

Digestive disorders and respiratory disease are the most frequently reported reasons for calf morbidity and mortality [3]. A recent study conducted on beef cattle farms in Canada [4] assessed the average herd-level treatment risk for preweaning calf diarrhea and bovine respiratory disease, and found these to be 4.9% and 3.0%, respectively. The same study estimated the average herd-level mortality within the first 24 hours of life, from 1 to 7 days, and from 7 days to weaning to be 2.3%, 1.1%, and 1.4%, respectively.

Morbidity and mortality in beef calves are associated with multiple infectious and noninfectious causes [5, 6], and are frequently concomitant with the failure of transfer of passive immunity (FTP). Recently, an assessment of the mean total costs associated with FTP in European systems pointed to €80 per beef calf (€20–139, in a 95% prediction interval) [7]. Although beef cows show considerable between-animal variance in first-milking colostrum yield and immunoglobulin concentration, research seems to show that factors influencing the time between birth and first suckling, including dam parity, udder and teat anatomy, and especially dystocia, are detrimental to calf passive immunity [8].

However, data on mortality and morbidity rates in extensive grazing beef cattle farms in Portugal are scarce, and mostly cover specific reproductive practices [9] or diseases [10, 11]. To the authors' knowledge, only one previous study published data on beef calf mortality in field conditions [12], and no studies have addressed its economic impacts. Hence, we conducted a preliminary study based on a questionnaire survey of farms in the Alentejo region, to assess the main farm management practices and to evaluate farmers' perception of how mortality and morbidity potentially affect production.

## 2. Materials and Methods

### 2.1. Questionnaire

Between April and June 2018, a questionnaire survey was conducted at 33 extensive beef cattle farms in North Alentejo, Portugal. Farmers were recruited at local livestock fairs and through contact with assisting veterinarians, thus representing a convenience sample. The objectives of the study were explained to the farmers, the confidentiality of individual data was assured, and their agreement with the publication of collected data was obtained. The addressed items included general feeding and reproduction management practices as well as quantitative data on the herd and the farmers' perception of the economic impacts of calf mortality and morbidity on their annual revenue. The questions in the questionnaire and the types of answers are described in Table 1. Following the collection of completed questionnaires, a data-sheet was built to allow for subsequent statistical analysis.

### 2.2. Statistical Analysis

The information regarding grazing area, herd size, and feeding and reproductive management (items 1–11 in Table 1) collected from the 33 questionnaires was used to present a general characterization of the sampled farms. The stocking rate was calculated by dividing the herd size (item 2) by the grazing area (item 1). As inconsistencies were found in quantitative answers (items 12–19 in Table 1) in 5 questionnaires, these were removed from further statistical analysis of data. Statistical analysis of data from the remaining 28 questionnaires was preceded by normality tests of variables (Shapiro–Wilks test); relevant variables exhibited distributions different from normality with positive skewness and were transformed using the function log10(*x*) or log10(*x* + 1) to achieve acceptable normality. Outliers were detected using Grubbs tests and were considered as missing data. Percent of weaned calves was calculated as the ratio between the number of weaned calves (item 13) and the number of live births (item 12). Correlation coefficients (Pearson's R) between transformed variables were also calculated. To detect statistical differences in the variables according to the farmers' perception of the economic impact of calf mortality, a one-way analysis of variance (ANOVA) was performed, followed by Duncan's multiple-range post hoc analysis. Finally, multiple regression models were tested for predictors of the number of calf deaths from birth to weaning, from birth to 30 days of age, and from 30 days to weaning. The chosen predictors were herd size and the number of cases of diarrhea, respiratory disease, and sudden deaths with no specific diagnosis in the last 12 months. Statistica v.12 software [13] was used for all statistical analyses.

## 3. Results

### 3.1. General Characterization of the Sampled Farms

**Figure 1:**
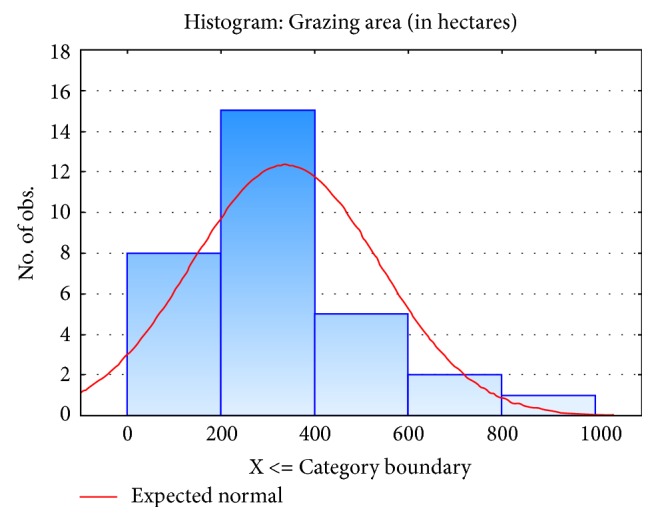
Frequency distribution of farms according to grazing area.

Farms were sampled in North Alentejo, Portugal, a region with a characteristic hot-summer Mediterranean climate (Csa, according to Köppen climate classification system) and typically with low organic matter content and low productivity soils [14]. The sizes of the farms included in the study ranged from 80 to 1000 hectares, with most farms having between 200 and 400 hectares (Figure 1). Herd size ranged from 27 to 411 animals, with 10 herds having 27–100 animals, 14 herds having 101–200 animals, and 9 herds having more than 201 animals.

The median herd size was 137 animals, and the median stocking rate was 0.41 adult animals/hectare. Only 18% of farms reared purebred indigenous breeds (namely, Alentejana and Mertolenga, the two most important indigenous beef cattle breeds in Alentejo) and 33% reared purebred exotic breeds (mostly Limousin, but Charolais in three farms), with the remaining 49% of farmers using crossbred cows as breeding stock (mostly crosses between Limousin and indigenous breeds). Thirteen farms reared livestock other than beef cattle (dairy cattle, equine, and/or pigs), but in all of them, beef cattle were the main source of revenue. In approximately half of the farms, bulls were kept with the herd all year round, while in the remaining farms, bulls stayed with the herd from late autumn to late spring, and were removed from the herd and kept in separate plots for the remaining period. Veterinary pregnancy diagnoses were performed in 21% of farms, estrus synchronization protocols were used in 18%, and artificial insemination protocols were used in 14%. All the farmers stated the need for supplementation of grazing with forage, or forage and concentrate, during the months when pasture was scarce, and the majority of farmers (70%) declared their farm was self-sufficient in forage. The geometric mean percent of weaned calves (defined as the number of weaned calves divided by the total number of live births) was 94.3% (leading to the assumption of 5.7% calf loss). In the previous 12 months, 78.8% of farms identified at least one case of calf diarrhea, 60.7% identified at least one case of respiratory disease, and 82.1% identified at least one sudden calf death, with no definitive diagnosis.

### 3.2. Association among Variables and the Effect of Perceived Economic Impact

As expected, positive correlations were found between herd size and calf deaths from birth to weaning, diarrhea, respiratory disease, and sudden deaths with no definitive diagnosis, but not with the percent of weaned calves. The percent of weaned calves negatively correlated with the number of deaths from birth to 30 days and the number of sudden deaths with no definite diagnosis. Mortality from birth to 30 days also positively correlated with the number of cases of diarrhea and respiratory disease (Table 2).

A one-way ANOVA showed significant differences in groups of farms, whose farmers had different perceptions of the economic impact of calf deaths on their annual revenue (Table 3) with regard to the number of cases of diarrhea and respiratory disease and the number of sudden deaths with no definitive diagnosis. Farmers who stated calf losses had a bigger impact on their annual revenue were from farms with a higher number of cases of sudden mortality and morbidity due to digestive and respiratory problems. A relatively high proportion (25%) of farmers were unable to assess the quantitative impact of calf losses on their revenue, but this group showed lower average values of the analyzed variables. We found no significant differences between groups regarding herd size, percent of weaned calves, and the number of calf deaths between birth and weaning.

We tested multiple regression models to explore the relevance of herd size and identified cases of diarrhea, respiratory disease, and sudden death as predictors of calf mortality rates. When considering calf deaths from birth to 30 days as the dependent variable (Table 4, adjusted *R*^2^ = 0.51, *p* = 0.000311), herd size was the only variable with statistical significance; the same was observed when the dependent variable was calf deaths from 30 days to weaning (Table 5, adjusted *R*^2^ = 0.38, *p* = 0.004481). Finally, when considering calf deaths from birth to weaning (Table 6, adjusted *R*^2^ = 0.67, *p* = 0.000306), the independent variable with statistical significance was the number of sudden deaths.

## 4. Discussion

### 4.1. General Characterization of the Sampled Farms

The median herd size (137 cows) was superior to the average herd size reported in the literature for the same region (55–80 animals, reported by Araújo et al. [14]). Stocking rate was also higher (0.41 vs. 0.34, reported by Carmona Belo et al. [12]), even though both values were similar to those reported by Milán et al. [15] under similar soil and climate conditions. The average herd size in cow-calf farms varies largely and may have an effect on revenue and on mortality and morbidity rates, depending on how the scale economy affects the herd size as well as how the herd size affects management intensity for optimizing production performance; the expected effect of herd size is, thus, nonlinear [16].

In general, the feeding and reproductive practices observed in our study were similar to those mentioned by Araújo et al. [14], even though some farms are now using reproductive monitoring and techniques, such as estrus synchronization and artificial insemination protocols, that were not traditionally used in extensive beef cattle systems in Portugal. This is probably a consequence of the recent changes in the country's rules for applying for funds that are now in line with the agricultural policies of the European Union, which since 2014 have made calving in the last 18 months a requirement for eligibility of suckling cows. The self-sufficiency of forage in the majority of farms is compliant with extensive production systems.

Carmona Belo et al. [12] reported a 3% calf mortality rate from birth to weaning in Alentejo cow-calf farms. In the present study, we assumed a higher rate of 5.7% based on the percent of weaned calves. This is a comparatively high rate, considering values reported in the literature (Table 7), even though the method of estimation may have influenced the result. The percent of farms with cases of calf diarrhea, respiratory disease, or sudden death without a definitive diagnosis in the last 12 months (78.8%, 60.7%, and 82.1%, respectively) was also considerably higher than that found in the literature (51.7% and 35.7% for calf diarrhea and sudden, unexplained deaths, respectively [17], and 21% for respiratory disease [5]). These results should be interpreted cautiously because the recruitment of respondents cannot exclude possible bias (i.e., farmers that responded to the questionnaire, namely, those recruited through the assisting veterinarians, could a priori own farms that had identified high morbidity and mortality rates). The fact that farms were not randomly chosen may have contributed to an artificial overestimation of the mortality and morbidity rates.

### 4.2. Association among Variables and the Effect of Perceived Economic Impact

We obtained moderate to strong positive correlations (0.43 ≤ *R* ≤ 0.73) between herd size and calf deaths from birth to weaning, as well as the detected number of cases of calf diarrhea, respiratory disease, and sudden, unexplained deaths. In dairy cattle farms, the reported associations of herd size and calf mortality found in the literature are contradictory, with some authors finding a positive association [28, 29], some a negative association [30, 31], and others finding no association between these variables [32]. In beef cattle, calf mortality rates increased with herd size, with significant differences among small (under 29 animals), medium (30–62 animals), and large herds [18]. Herd size can influence the density of pathogens in the environment if the pasture area is limited (hence, the stocking rate is high) [18]. Furthermore, the accumulation of calvings may create favorable conditions for infection spread, together with the lack of dedicated human care and available resources, thereby leading to higher mortality rates [3]. A larger herd size has previously been associated with less intensive calving management [18]. However, studies wherein the herd size did not significantly affect calf mortality, despite the number of calves cared per operator being higher in larger herds than in smaller ones, have suggested better specialization of the workers in larger herds [32]. Considering that, in the Alentejo region, extensive cow-calf farming systems usually depend on a reduced number of low skilled workers, and that herd size in the sampled farms corresponds to medium to large herds, it is possible that the relatively high mortality rates are associated with poor calf management practices.

Significant correlations were observed between the detected number of cases of calf diarrhea, respiratory disease, and sudden, unexplained deaths. Studies have shown that perinatal diarrhea may predispose calves to respiratory disease [5, 18]. Additionally, calf diarrhea pathogens have been associated with sudden death in calves, mainly in the period from birth to 14 days, during which death can occur quickly [17], sometimes in a matter of hours, without the calves necessarily exhibiting easily identifiable symptoms [33]. This suggests that the number of cases of calf diarrhea detected by farmers can be underestimated. The detected number of sudden deaths also positively correlated with calf mortality, both from birth to 30 days and from 30 days to weaning. Sudden deaths in calves can have infectious causes (namely, neonatal septicemia due to failure of passive transfer and exposure to a virulent bacterium, such as *Escherichia coli* or *Salmonella* sp., or later onset septicemia due to virulent strains of *Salmonella* sp. or *Mannheimia haemolytica* [34]) or noninfectious causes, such as trauma or abomasal ulcers due to poor feeding management or environmental stress [35]. Regardless of the cause, a high incidence of sudden calf deaths can indicate the need for improved management strategies.

In young beef calves, diarrhea, and respiratory disease are the two most common causes of mortality [3], accounting for approximately 50% and 15% of all calf deaths, respectively [29]. Calf diarrhea is particularly important as a cause of death from birth to 14 days, while respiratory disease often occurs later in the preweaning period [18]. Reported on-farm mortality rates were higher between birth and 3 months of age, and declined considerably between 3 months and weaning [36]. In our study, a significant correlation was observed between mortality between birth and 30 days and mortality from 30 days to weaning; mortality between birth and 30 days positively correlated with the number of detected cases of diarrhea and respiratory disease, but mortality from 30 days to weaning did not present significant correlations with either variable.

Infectious diarrhea is one of the biggest health challenges in the beef cattle industry, as more than 20% of beef cattle owners state that calf diarrhea has a significant impact on their economic productivity [37]. Respiratory disease in nursing calves also represents an important source of loss for some cow-calf producers, but the lack of published research on risk factors makes it difficult for veterinarians to make evidence-based decisions regarding practices to minimize the disease in affected operations [38]. In this study, the number of detected cases of both diseases, as well as of sudden, unexplained deaths, were significantly different in farms with lower and higher assessments of farmers of the economic impact of calf death on annual revenue. Surprisingly, one-quarter of the farmers were unable to estimate this impact. The fact that farmers are unaware of or oblivious to the economic impacts of nursing calf morbidity and deaths on their farms probably has a negative effect over the willingness to adopt practices to mitigate this impact, with repercussions both on economic viability and animal welfare.

In the multiple regression models for calf deaths from birth to 30 days and from 30 days to weaning, herd size was a predictor with a statistically significant effect. As pointed out earlier, a larger herd size can represent a negative effect over mortality risk, since bigger herds need a larger availability of human resources to effectively monitor and intervene, when necessary, in events that can lead to calf loss, if not attended to fast enough. This can pose an even more serious problem when the calving season is concentrated in a specific period of the year, because the necessary addition to the workforce may not be met by the farmers, who usually try to minimize costs on human resources, thereby increasing the number of calf losses due to the lack of timely human care. When the regression model included calf deaths from birth to weaning as a dependent variable, the only predictor with a statistically significant value was the number of sudden deaths with no specific diagnosis. As stated previously, this suggests an influence of unmeasured management variables over calf mortality that may be influencing the causes of calf losses in both periods.

As stated by Murray et al. [4], it is important to acknowledge that the herd‐level incidence of mortality and disease was estimated by respondents and that farmers' perception and recollection of events can lead to response and recall biases, with respondents either intentionally misrepresenting or unintentionally lacking accuracy in their responses. As this study was based on a limited number of farms, the results should be interpreted cautiously. Nevertheless, the results seem to suggest that mortality and morbidity levels in extensive cow-calf beef herds in the Alentejo region are high, with potentially serious impacts on both animal welfare and farm sustainability.

## 5. Conclusions

In this preliminary study on extensive beef cattle farms in Alentejo, Portugal, the reported calf mortality rates were higher than those reported in similar studies in other parts of the world. The number of cases of diarrhea, respiratory disease, and sudden death was also comparatively high. This can potentially mean that there are health and welfare issues that need improvement; nonetheless, the results should be interpreted cautiously, taking into consideration the small number of farms included in the study. Surprisingly, a considerable number of farmers seem unaware of the economic impact of calf morbidity and mortality on their farm's revenue. Herd size showed some predictive value on calf mortality during the first month of life and from 30 days to weaning. The number of sudden, unexplained deaths can also help predict calf mortality in the overall birth to weaning period. Broader studies are needed to better understand management practices and other factors that may be affecting these results, so that measures can be taken to mitigate their impacts on animal welfare and farm sustainability.

## Figures and Tables

**Table 1:** Questionnaire items and types of required answers.

Number	Question	Answer
1	Grazing area	Number (hectares)
2	Herd size	Number (breeding cows in the last 12 months)
3	Breed(s)	Multiple choices (list of beef cattle breeds, crosses)
4	Other livestock	Multiple choices (equine, dairy cattle, sheep, pigs)
5	Main revenue source	Beef cattle/other
6	Self-sufficiency in forage in a typical year	Yes/no
7	Supplements in periods of scarcity	Yes/no; if yes, multiple-choice (forage, concentrate, both)
8	Management of bulls in the herd	All year round/restricted period
9	Pregnancy diagnosis	Yes/no
10	Synchronization of estrus	Yes/no
11	Artificial insemination	Yes/no
12	Births	Number (live births in the last 12 months)
13	Weaned calves	Number (weaned calves in the last 12 months)
14	Calf deaths from birth to 30 days	Number (deaths in the last 12 months)
15	Calf deaths from 30 days to weaning	Number (deaths in the last 12 months)
16	Cases of sudden calf death, undiagnosed	Number (deaths in the last 12 months)
17	Cases of calf diarrhea	Number (cases in the last 12 months)
18	Cases of calf respiratory disease	Number (cases in the last 12 months)
19	Estimation of the economic impact of calf deaths	Multiple choice (unknown, under 2%, 2%–5%, 5%–10%, over 10%)

**Table 2:** Pearson's correlation coefficients among variables (*n* = 28).

	Herd size	% Weaned calves	Deaths (birth to 30 d)	Deaths (30 d to weaning)	Diarrhea	Respiratory disease	Sudden deaths
Herd size	1.00	N.S.	0.73	0.61	0.65	0.43	0.61
% Weaned calves	N.S.	1.00	−0.51	N.S.	N.S.	N.S.	−0.42
Deaths (birth to 30 d)	0.73	−0.51	1.00	0.66	0.47	0.38	0.62
Deaths (30 d to weaning)	0.61	N.S.	0.66	1.00	N.S.	N.S.	0.54
Diarrhea	0.65	N.S.	0.47	N.S.	1.00	0.59	0.52
Respiratory disease	0.43	N.S.	0.38	N.S.	0.59	1.00	0.46
Sudden deaths	0.61	−0.42	0.62	0.54	0.52	0.46	1.00

Herd size and % weaned calves were log10(*x*) transformed; the remaining variables were log10(*x* + 1) transformed; values are significant at the *p* < 0.05 level; N.S., not significant.

**Table 3:** Effect of farmers' perception of the economic impact of calf death on annual revenue (ANOVA and Duncan's post hoc test) (least-square mean ± standard errors).

Perception of the economic impact on annual revenue	Unknown (*n* = 7)	Under 2% (*n* = 12)	2–5% (*n* = 4)	5–10% (*n* = 2)	Over 10% (*n* = 3)	*p*-Value
Herd size	2.03 ± 0.12	2.04 ± 0.09	1.81 ± 0.15	2.39 ± 0.22	2.24 ± 0.18	0.220
% Weaned calves	−0.02 ± 0.01	−0.02 ± 0.01	−0.04 ± 0.01	−0.03 ± 0.02	−0.04 ± 0.01	0.492
Deaths (birth to 30 d)	0.52 ± 0.12	0.48 ± 0.09	0.24 ± 0.15	0.52 ± 0.22	0.91 ± 0.18	0.119
Deaths (30 d to weaning)	0.48 ± 0.12	0.26 ± 0.09	0.15 ± 0.16	0.52 ± 0.22	0.46 ± 0.18	0.355
Diarrhea	0.37 ± 0.15^a^	0.43 ± 0.11^a^	0.29 ± 0.19^a^	1.22 ± 0.27^b^	1.28 ± 0.22^b^	0.003
Respiratory disease	0.09 ± 0.10^a^	0.38 ± 0.08^ab^	0.35 ± 0.13^ab^	0.76 ± 0.18^b^	0.76 ± 0.15^b^	0.005
Sudden deaths	0.39 ± 0.10^ab^	0.18 ± 0.08^a^	0.15 ± 0.13^a^	0.87 ± 0.19^c^	0.68 ± 0.15^bc^	0.005

Herd size and % weaned calves were log10(*x*) transformed; the remaining variables were log10(*x* + 1) transformed; different letters in superscript correspond to statistically different means; values are significant at the *p* < 0.05 level.

**Table 4:** Multiple regression model for calf deaths from birth to 30 days (*n* = 28).

	b∗	Std. Err. of b∗	b	Std. Err. of b	*p*-Value
Intercept	—	—	−0.86	0.37	0.029
Herd size	0.60	0.20	0.63	0.20	0.005
Diarrhea	−0.10	0.20	−0.07	0.13	0.612
Respiratory disease	0.05	0.17	0.05	0.17	0.768
Sudden deaths	0.28	0.18	0.28	0.18	0.127

b, coefficient; Std. Err., standard error; b∗, standardized coefficient; values are significant at the *p* < 0.05 level.

**Table 5:** Multiple regression model for calf deaths from 30 days to weaning (*n* = 28).

	b∗	Std. Err. of b∗	b	Std. Err. of b	*p*-Value
Intercept	—	—	−0.83	0.40	0.050
Herd size	0.56	0.22	0.58	0.22	0.016
Diarrhea	−0.17	0.23	−0.11	0.15	0.466
Respiratory disease	−0.18	0.19	−0.19	0.19	0.351
Sudden deaths	0.35	0.20	0.34	0.19	0.092

b, coefficient; Std. Err., standard error; b∗, standardized coefficient; values are significant at the *p* < 0.05 level.

**Table 6:** Multiple regression model for calf deaths from birth to weaning (*n* = 28).

	b∗	Std. Err. of b∗	b	Std. Err. of b	*p*-Value
Intercept	—	—	−0.48	0.46	0.308
Herd size	0.31	0.18	0.40	0.24	0.108
Diarrhea	0.07	0.18	0.05	0.13	0.725
Respiratory disease	0.13	0.16	0.14	0.17	0.420
Sudden deaths	0.51	0.16	0.54	0.17	0.005

b, coefficient; Std. Err., standard error; b∗, standardized coefficient; values are significant at the *p* < 0.05 level.

**Table 7:** Reported mortality rates in beef calves.

Author	Country	Description of variable	Mortality rate
Bleul [18]	Switzerland	Birth to 120 days	4.8%
Carmona Belo et al. [12]	Portugal	Birth to weaning	3%
Cervantes et al. [19]	Spain	Birth to weaning	9.6%
Guerra et al. [20]	United States	Assumed from calf survival rate from birth to weaning of 91%, in LA state	9%
Kamali et al. [21]	Brazil	Birth to weaning	4%
Mõtus et al. [3]	Estonia	1–5 months	2.9%
Ortiz-Pelaez et al. [22]	United Kingdom	Mortality per 180 days, in 3 different regions	1.8%–6%
Perrin et al. [23]	France	7 days–6 months	6.5%
Ramsay et al. [16]	United States	Birth to weaning, in 3 states (TX, OK, and NM)	3.5%
Ring et al. [24]	Ireland	3 days–6 months	6.7%
Sanderson and Dargatz [25]	United States	Birth to weaning	3.7%
Todd et al. [26]	Ireland	Birth to 6 months, at 2 different levels	4.7% and 6%
Waldner et al. [27]	Canada	Birth to 3 months	3.3%

## Data Availability

The survey data used to support the findings of this study are available from the corresponding author upon request.

## References

[1] GPP GPP Market information 2017—Animal products. GPP—Gabinete de Planeamento, Políticas e Administração Geral | Office for Planning, Policies, and Administration. http://www.gpp.pt/images/GPP/O_que_disponibilizamos/Publicacoes/Periodicos/InfoMercados_ProdutosAnimais_Final.pdf.

[2] INE Statistics Portugal (2018). *Statistical Yearbook of Alentejo Region 2017*.

[3] Mõtus K., Viltrop A., Emanuelson U. (2018). Reasons and risk factors for beef calf and young stock on-farm mortality in extensive cow-calf herds. *Animal*.

[4] Murray C. F., Fick L. J., Pajor E. A., Barkema H. W., Jelinski M. D., Windeyer M. C. (2016). Calf management practices and associations with herd-level morbidity and mortality on beef cow-calf operations. *Animal*.

[5] Wooloums A. R., Berghaus R. D., Smith D. R. (2013). Producer survey of herd-level risk factors for nursing beef calf respiratory disease. *Journal of the American Veterinary Medical Association*.

[6] Mawatari T., Hirano K., Ikeda H., Tsunemitsu H., Suzuki T. ( 2014). Surveillance of diarrhea-causing pathogens in dairy and beef cows in Yamagata Prefecture, Japan from 2002 to 2011. * Microbiology and Immunology*.

[7] Raboisson D., Trillat P., Cahuzac C. (2016). Failure of passive immune transfer in calves: a meta-analysis on the consequences and assessment of the economic impact. *PLoS One*.

[8] McGee M., Earley B. (2019). Review: passive immunity in beef-suckler calves. *Animal*.

[9] Quaresma M. A., da Costa L. L., Horta A. E. M., Silva J. R. (2004). Twinning induction and its effects on embryo-foetal and calf survival, and on reproductive efficiency of Mertolengo cattle kept at pasture. *Revista Portuguesa de Ciências Veterinárias*.

[10] Stilwell G., Matos M., Carolino N., Lima M. S. (2008). Effect of a quadrivalent vaccine against respiratory virus on the incidence of respiratory disease in weaned beef calves. *Preventive Veterinary Medicine*.

[11] Branco S., Orvalho J., Leitão A. (2010). Fatal cases of *Theileria annulata *infection in calves in Portugal associated with neoplastic-like lymphoid cell proliferation. *Journal of Veterinary Science*.

[12] Carmona Belo C., Belo A. T., Felício N., Martins J., Domingos T. (2013). Parâmetros reprodutivos de efetivos de vacas aleitantes no Alentejo. *Revista das Ciências Agrárias*.

[13] StatSoft (Inc.) (April 2019). Statistica software, v. 12.0. https://www.tibco.com/products/data-science.

[14] Araújo J. P., Cerqueira J., Vaz P. S., Pinto de Andrade L., Várzea Rodrigues J., Rodrigues A. M. Extensive beef cattle production in Portugal.

[15] Milán M. J., Bartolomé J., Quintanilla R., García-Cachán M. D., Espejo M., Herráiz P. L., Sánchez-Recio J. M., Piedrafita J. (2006). Structural characterization and typology of beef cattle farms of Spanish wooded rangelands (dehesas). *Livestock Science*.

[16] Ramsay R., Doye D., Ward C., McGrann J., Falconer L., Bevers S. (2005). Factors affecting beef-cow herd costs, production, and profits. *Journal of Agricultural and Applied Economics*.

[17] Lievaart J. J., Charman N. R., Scrivener C., Morton A., Allworth M. B. (2013). Incidence of calf scours and associated risk factors in southern New South Wales beef herds. *Australian Veterinary Journal*.

[18] Bleul U. (2011). Risk factors and rates of perinatal and postnatal mortality in cattle in Switzerland. *Livestock Science*.

[19] Cervantes I., Gutiérrez J. P., Fernández I., Goyache F. (2010). Genetic relationships among calving ease, gestation length, and calf survival to weaning in the Asturiana de los Valles beef cattle breed. *Journal of Animal Science*.

[20] Guerra J. L., Franke D. E., Blouin D. C. (2006). Genetic parameters for calving rate and calf survival from linear, threshold, and logistic models in a multibreed beef cattle population. *Journal of Animal Science*.

[21] Pashaei Kamali F., van der Linden A., Meuwissen M. P. M., Malafaia G. C., Oude Lansink A. G. J. M., de Boer I. J. M. (2016). Environmental and economic performance of beef farming systems with different feeding strategies in southern Brazil. *Agricultural Systems*.

[22] Ortiz-Pelaez A., Pritchard D. G., Pfeiffer D. U., Jones E., Honeyman P., Mawdsley J. J. (2008). Calf mortality as a welfare indicator on British cattle farms. *The Veterinary Journal*.

[23] Perrin J.-B., Ducrot C., Vinard J.-L., Hendrikx P., Calavas D. (2011). Analyse de la mortalité bovine en France de 2003 à 2009. *INRA Productions Animales*.

[24] Ring S. C., McCarthy J., Kelleher M. M., Doherty M. L., Berry D. P. (2018). Risk factors associated with animal mortality in pasture-based, seasonal-calving dairy and beef herds. *Journal of Animal Science*.

[25] Sanderson M. W., Dargatz D. A. (2000). Risk factors for high herd level mortality risk from birth to weaning in beef herds in the USA. *Preventive Veterinary Medicine*.

[26] Todd C. G., McGee M., Tiernan K. (2018). An observational study on passive immunity in Irish suckler beef and dairy calves: tests for failure of passive transfer of immunity and associations with health and performance. *Preventive Veterinary Medicine*.

[27] Waldner C., Jelinski M. D., McIntyre-Zimmer K. (2013). Survey of western Canadian beef producers regarding calf-hood diseases, management practices, and veterinary service usage. *The Canadian Veterinary Journal*.

[28] Gulliksen S. M., Lie K. I., Løken T., Østerås O. (2009). Calf mortality in Norwegian dairy herds. *Journal of Dairy Science Home*.

[29] Uetake K. (2013). Newborn calf welfare: a review focusing on mortality rates. *Animal Science Journal*.

[30] Raboisson D., Delor F., Cahuzac E., Gendre C., Sans P., Allaire G. (2013). Perinatal, neonatal, and rearing period mortality of dairy calves and replacement heifers in France. *Journal of Dairy Science Home*.

[31] Jago J. G., Berry D. P. (2011). Associations between herd size, rate of expansion and production, breeding policy and reproduction in spring-calving dairy herds. *Animal*.

[32] Zucali M., Bava L., Tamburini A., Guerci M., Sandrucci A. (2013). Management risk factors for calf mortality in intensive Italian dairy farms. *Italian Journal of Animal Science*.

[33] Simpson K. M., Callan R. J., Van Metre D. C. (2018). Clostridial abomasitis and enteritis in ruminants. *Veterinary Clinics of North America: Food Animal Practice*.

[34] Fecteau G., Smith B. P., George L. W. (2009). Septicemia and meningitis in the newborn calf. *Veterinary Clinics of North America: Food Animal Practice*.

[35] Marshall T. S. (2009). Abomasal ulceration and tympany of calves. *Veterinary Clinics of North America: Food Animal Practice*.

[36] Mõtus K., Reimus K., Orro T., Viltrop A., Emanuelson U. (2017). On‐farm mortality, causes and risk factors in Estonian beef cow-calf herds. *Preventive Veterinary Medicine*.

[37] Foster D. M., Smith G. W. (2009). Pathophysiology of diarrhea in calves. *Veterinary Clinics of North America: Food Animal Practice*.

[38] Woolums A. R., Berghaus R. D., Smith D. R. (2018). Case-control study to determine herd-level risk factors for bovine respiratory disease in nursing beef calves on cow-calf operations. *Journal of the American Veterinary Medical Association*.

